# Impact of Gadolinium on the Structure and Magnetic Properties of Nanocrystalline Powders of Iron Oxides Produced by the Extraction-Pyrolytic Method

**DOI:** 10.3390/ma13184147

**Published:** 2020-09-17

**Authors:** Vera Serga, Regina Burve, Mikhail Maiorov, Aija Krumina, Ramūnas Skaudžius, Aleksej Zarkov, Aivaras Kareiva, Anatoli I. Popov

**Affiliations:** 1 Institute of Solid State Physics, University of Latvia, Kengaraga 8, LV-1063 Riga, Latvia; vera_serga@inbox.lv (V.S.); regina.burve@cfi.lu.lv (R.B.); popov@latnet.lv (A.I.P.); 2 Institute of Inorganic Chemistry, Riga Technical University, P. Valdena 3/7, LV-1048 Riga, Latvia; aija.krumina@rtu.lv; 3 Institute of Physics, University of Latvia, Miera 32, LV-2169 Salaspils, Latvia; maiorov@sal.lv; 4 Institute of Chemistry, Vilnius University, Naugarduko st. 24, LT-03225 Vilnius, Lithuania; ramunas.skaudzius@chgf.vu.lt (R.S.); aleksej.zarkov@chf.vu.lt (A.Z.); 5 Institute of Physics, University of Tartu, W. Ostwald Str. 1, 50411 Tartu, Estonia

**Keywords:** iron oxides, nanostructures, gadolinium impact, extraction–pyrolitic method, magnetization, coercivity

## Abstract

Interest in magnetic nanoparticles is primarily due to their practical use. In this work, for the production of nanocrystalline powders of pure and gadolinium doped iron oxides, the extraction-pyrolytic method (EPM) was used. As a precursor, either iron-containing extract (iron (III) caproate in caproic acid) or its mixture with gadolinium-containing extract (gadolinium (III) valerate in valeric acid) was used. The mixed precursor contained 0.5 mol %, 2.5 mol %, 12.5 mol %, 50 mol %, and 75 mol % gadolinium in relation to the iron content. The formation of iron oxide phases, depending on the preparation conditions, was investigated. According to the results obtained, it was demonstrated that the presence of more than 2.5 mol % gadolinium additive in the mixed precursor inhibits the magnetite-to-hematite transformation process during thermal treatment. Produced samples were characterized by XRD and SEM methods, and the magnetic properties were studied.

## 1. Introduction

Magnetic nanoparticles play an important role in the rapidly developing branches of science focusing on the study of nanocrystalline materials. Among all magnetic phases of iron oxides, maghemite (γ-Fe_2_O_3_) and magnetite (Fe_3_O_4_) stand out due to strong magnetic moments and some structural features. Magnetite has the inverse spinel structure, in which half of Fe^3+^ ions occupy tetrahedral positions, while Fe^3+^ and Fe^2+^ are uniformly distributed over octahedral positions. Maghemite also has the inverse spinel structure, along with structural vacancies. However, unlike magnetite, it contains only Fe^3+^ ions [1]. Hematite (α-Fe_2_O_3_) is another phase of iron oxide, in which all Fe^3+^ ions have an octahedral coordination. It is antiferromagnetic at temperatures below 950 K, while, above 260 K, it exhibits so-called “weak” ferromagnetism [2,3]. In air, magnetite-to-hematite oxidation proceeds either directly or via maghemite [4,5]. 

Iron oxides are widely used as magnetic pigments in electronic recording devices and as catalysts in such industrially important synthesis as the Haber Process for the synthesis of NH_3_, the Fischer-Tropsch synthesis of hydrocarbons, and the water gas shift (WGS) reaction for the hydrogen production [6]. Active research is being carried out on the use of iron oxides as heterogeneous Fenton-like catalysts for the oxidative treatment of polluted water [5,6,7]. As with other important applications, adsorbents for water and gas purification, and starting material in ferrofluid production and in medicine can be mentioned [6,8,9,10,11,12]. The introduction of rare earth elements, such as gadolinium (III) ions into the structure of magnetite nanoparticles, can improve magnetic and optical properties, and some limitations for biomedical applications can be overcome [13].

Currently, the most common method for producing nanosized particles of magnetite is the coprecipitation of a mixture of iron salts (Fe^2+^ and Fe^3+^) with an alkaline agent in either air or an inert atmosphere, at room or elevated temperature. Oleic acid is usually used as a particle stabilizer [14,15]. In addition, such methods as sol-gel [16,17], hydrothermal [18], microemulsion [19], etc., are also used to produce magnetic nanoparticles. To produce Gd-doped magnetite nanoparticles, such chemical synthetic routes have been employed as co-precipitation [20,21], polyol synthesis [22], and hydrothermal [23], as well as the thermal decomposition of a mixture of iron and gadolinium acetylacetonates [24].

The introduction of various amounts of metal cations, including Gd^3+^, during the synthesis of iron oxides, affects the phase composition, shape and size of particles, as well as the properties of the produced iron oxides [25,26,27]. It was shown [4] that the maghemite-to-hematite transformation temperature varied from 500 °C for pure maghemite to 540–650 °C for less than 1% metal-containing maghemite (metals—Co, Ni, Zn, Cu, Mn, A1, V, and Cr).

In the preparation of nanosized oxide materials, metal alkoxides are widely used as precursors. The second most commonly used class of compounds are metal carboxylates [28]. In the extraction–pyrolytic method (EPM), saturated fatty acids (with or without diluent) are used as an extractant to produce metal-containing extracts (precursors)—solutions of metal carboxylates in carboxylic acid or diluent, followed by heat treatment (pyrolysis) [29]. Moreover, within the framework of the method, the desired ratio of elements is preserved in the final product, since aliquots of solutions with the established concentration of metals are mixed. The method does not require high-temperature treatment, toxic reactants, complex operations, or special equipment. Using EPM, various oxide materials are produced, including nanocrystalline powders of cobalt Co_3_O_4_, tungsten WO_3_, gadolinium Gd_2_O_3_ and magnesium MgO oxides [30,31,32,33], supported catalysts [34], and magnetic materials, such as FePt and Fe_3_O_4_/Pt [35,36]. The performed investigations showed that this synthesis method is fast, scalable, and well reproducible, providing a promising avenue for industrial applications of novel magnetic nanoparticles.

The aim of this work is to investigate the formation of iron oxide phases depending on the pyrolysis temperature of the iron(III)-containing extract (precursor) and the presence of different amounts of gadolinium (III) additive, as well as to investigate the magnetic properties of all produced materials.

## 2. Materials and Methods 

Organic metal-containing extracts (precursors) were prepared by liquid–liquid extraction. The general equation for the extraction of trivalent metal cations with fatty acid HR can be represented as: Me^3+^ + (3+s)HR + qH_2_O ↔ [MeR_3_·sHR]·qH_2_O + 2H^+^ [37], where s and q—solvation and hydration numbers, respectively. The extraction of iron ions from a 1 M aqueous solution of Fe(NO_3_)_3_ was carried out using caproic acid (C_5_H_11_COOH) as an extractant, without diluent and with the addition of stoichiometric amounts of 1 M NaOH solution to obtain the desired amount of Fe in organic phase. The initial ratio of the volume of aqueous and organic phases in the extraction system was 1:1. After the completion of the extraction, the pH of the aqueous phase was ~0.9. To remove small amount of co-extracted sodium ions, the organic phase (extract) was washed five times with a freshly prepared 1 M solution of Fe(NO_3_)_3_. In this way, precursor 1—a solution of iron caproate in caproic acid—was prepared. To establish the iron concentration in precursor 1, the gravimetric method for the determination of iron in the form of Fe_2_O_3_ was used [38]. Heat treatment of the pyrolysis product of the precursor aliquot at 900 °C was performed several times until a constant mass of the sample was achieved. In accordance with the obtained results, the iron concentration was established to be C_Fe_ = 0.69 M. To prepare gadolinium-containing extract, precursor 2, valeric acid (C_4_H_9_COOH) without diluent was used as an extractant. The procedure of producing a solution of gadolinium valerate in valeric acid is described in detail in [32]. The gadolinium concentration was established to be 0.50 M. To study the effect of the presence of gadolinium on the phase composition of decomposition products of precursor 1, a mixture of precursors 1 and 2 was used. The mixed precursor contained 0.5 mol %, 2.5 mol %, 12.5 mol %, 50 mol %, and 75 mol % gadolinium in relation to the iron content. In order to produce nanocrystalline powders, heat treatment (pyrolysis) of precursor 1 or a mixture of precursors 1 and 2 was carried out in a porcelain crucible by heating the air from room temperature (T) to 350–550 °C at a heating rate of 10 °C/min and annealing (t_anneal_) for 30–60 min, followed by rapid cooling in ambient conditions. Pyrolysis was performed in a laboratory furnace SNOL 8.2/1100 (furnace working chamber dimensions 195 mm × 310 mm × 135 mm). Produced samples were then ground by a pestle in agate mortar and collected. Further investigations were used only with as-prepared powders without any additional post-treatment. 

The thermal stability of the extract was studied in a static air atmosphere by thermogravimetric analysis (TGA) and high temperature differential scanning calorimetry (HDSC) (LINSEIS STA PT 1600, Selb, Germany). The sample was heated from room temperature to 700 °C in a static air atmosphere at a heating rate of 5 °C/min. The phase composition of produced materials was determined by X-ray diffraction method (XRD), using a diffractometer D8 Advance (Bruker) with copper anode x-ray lamp (CuKα radiation, λ = 1.5418 Å, 1.6 kW, Karlsruhe, Germany) in a wide range of Bragg angles (10° < 2θ < 75°), with a scanning rate of 0.02°/s at room temperature. XRD patterns were referenced to the PDF ICCD 01-071-6336 (Fe_3_O_4_), 00-039-1346 (γ-Fe_2_O_3_), 01-087-1165 (α-Fe_2_O_3_), and 00-012-0797 (Gd_2_O_3_). The mean crystallite size *d* of iron oxides was calculated using the Scherrer method for the most intense peaks at the planes (311) and (104) for Fe_3_O_4_ and α-Fe_2_O_3_, respectively. Magnetic measurements were carried out at room temperature, using a vibrating sample magnetometer (Lake Shore Cryotronics, Inc., model 7404, Westerville, OH, USA) with a maximum magnetic field of 10 kOe. SEM measurements were made using the Tescan Lyra-3, which was equipped with an Energy Dispersive Spectrometer (EDS) for composition analysis. Investigations were performed with pre-applied gold coating (~20 nm layer) on the surface of samples.

## 3. Results and Discussion

**Figure 1:**
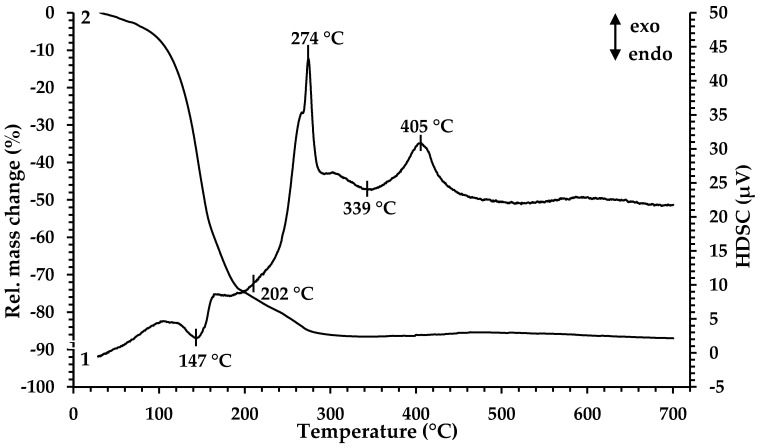
Thermal analysis of Fe-containing extract (precursor 1): 1—HDSC, 2—TG.

For determination of the minimal temperature for organic component removal and extract decomposition, TGA and HDSC were performed. According to the results presented in Figure 1, the thermal decomposition of the Fe-containing extract proceeds in four main stages at temperatures of 147 °C, 202 °C, 274 °C and 339 °C. 

The decrease in the mass of the sample (~20%) begins when the sample is heated from room temperature to 125 °C. In this case, noticeable thermal effects on the HDSC curve are not observed. The first endothermic peak in the temperature range 125–161 °C is accompanied by a significant weight loss of the sample (~39%). The mass loss in this temperature range is associated with the evaporation of coextracted water and free extractant (caproic acid). At 168 °C, a wide endothermic peak begins; it ends at 238 °C and is accompanied by a weight loss of ~21%. Then, in a narrow temperature range of 261–282 °C, there is a sharp exothermic peak, at which the mass loss is ~6%. The processes occurring in these temperature ranges are directly related to the thermal decomposition of iron caproate, occurring with the formation of intermediate compounds and followed by their thermal degradation. At the first stage, Fe^3+^ ions are reduced to Fe^2+^ with the formation of iron (II) caproate [39], as well as decarboxylation reaction (elimination of CO_2_) with the formation of ketone and metal carbonate, which is specific for the thermal decomposition reaction of metal carboxylates [40,41]. At the second stage, ketone oxidation and the decomposition of iron (II) carbonate occur, resulting in the formation of mixed Fe_3_O_4_ oxide. Then, a wide endothermic peak on the HDSC curve at a temperature of 315 °C appears which is associated with the removal of gaseous oxidation products. The process is characterized by a mass loss of ~1%. The exothermic peak observed at the temperature range 381–429 °C (peak maximum at 405 °C) corresponds to a slight increase in the sample mass (~1%) associated with the oxidation of Fe^2+^ to Fe^3+^ in Fe_3_O_4_ by atmospheric oxygen and the following formation of Fe_2_O_3_. Based on the results obtained, the temperature 350 °C was chosen as the minimum pyrolysis temperature for precursor 1. According to the results presented in [32], during the heat treatment of precursor 2, the mass loss of the sample ends at 450 °C, and upon reaching 550 °C, the crystallization of gadolinium oxide begins. Therefore, a mixture of precursors 1 and 2 was processed at 550 °C.

Table 1 summarizes the main results of XRD analysis and magnetic measurements of the powders produced in the framework of this study. Furthermore, the similarity of the crystal lattices of Fe_3_O_4_ and γ−Fe_2_O_3_ [1,36] does not allow us to conclude which magnetic phase is present in the produced samples without additional investigations.

**Figure 2:**
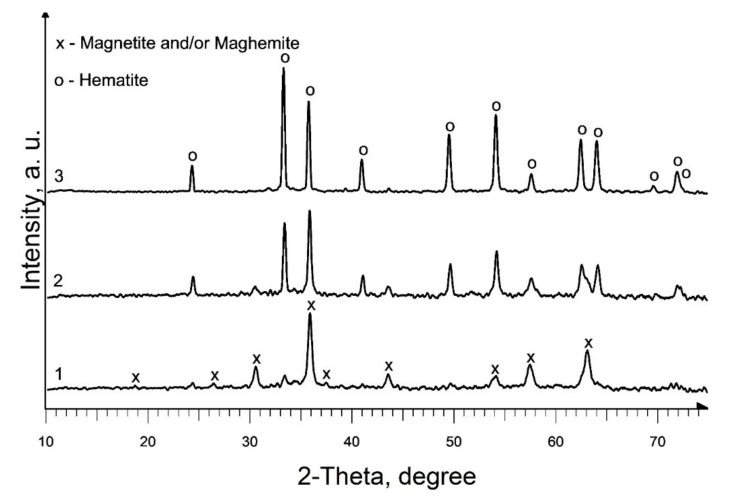
XRD patterns of produced pure iron oxides samples: 1—S1; 2—S2; 3—S3.

The phase composition of the powder produced as a result of heat treatment of precursor 1, at a temperature of 350 °C (Figure 2, curve 1; Table 1, S1), is identical to the phase composition of the pyrolysis products of individual iron (III) caproate produced in [36]: main magnetic phase—magnetite (Fe_3_O_4_) and maghemite (γ-Fe_2_O_3_) and a non-magnetic admixture of hematite (α-Fe_2_O_3_). Moreover, the predominant content of magnetite in the magnetic phase was confirmed by magnetic measurements. An increase in the pyrolysis temperature to 450 °C leads to an increase in the content of the hematite phase and an increase in *d* of iron oxides in the produced material (Figure 2, curve 2; Table 1, S2). According to XRD data, as a result of pyrolysis at 550 °C, a single-phase sample of α-Fe_2_O_3_ is formed (Figure 2, curve 3; Table 1, S3). A double increase in the duration of annealing at a given temperature results in only a slight increase in the *d* of hematite (Table 1, S3 and S4).

**Figure 3:**
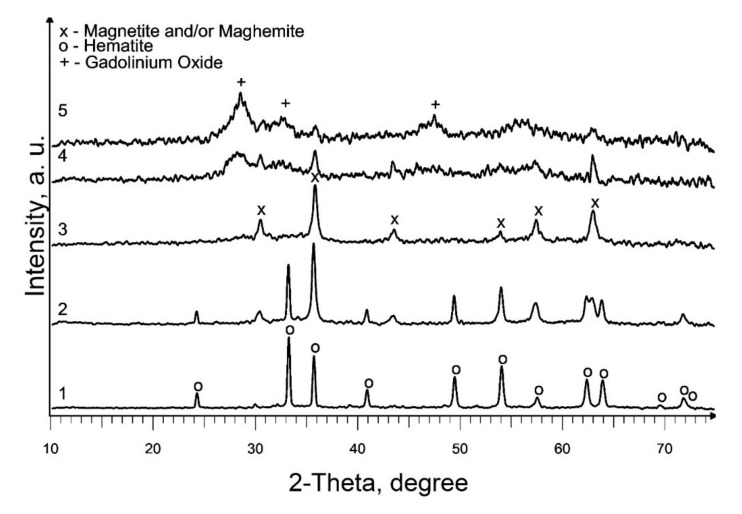
XRD patterns of produced Gd-containing iron oxides samples: 1—S5; 2—S6; 3—S7; 4—S8; 5—S9.

Based on the results acquired, studies on the effect of the addition of precursor 2 (a solution of gadolinium (III) valerate in valeric acid) on the phase composition of the end products of pyrolysis of precursor 1 (a solution of iron (III) caproate in caproic acid) were carried out at 550 °C. According to the XRD results, the composition of the produced samples depends on the gadolinium content in the initial mixture (Figure 3; Table 1, S5–S9). Thus, the presence of 0.5 mol % gadolinium has practically no effect on the phase composition of the final product (Figure 2, curve 3 and Figure 3, curve 1). With an increase in the gadolinium content to 2.5 mol %, along with the main crystalline phase of hematite, an admixture magnetic phase (magnetite and/or maghemite) appears, and at 12.5 mol % only a magnetic phase is present in the sample (Figure 3, curves 2 and 3). So, in the first case, the inhibition of maghemite-to-hematite transformation is observed, but in the second, this transformation is not observed. Moreover, a further increase in gadolinium content to the molar ratio Gd:Fe = 1:1 and Gd:Fe = 3:1 in mixed precursors leads to a formation of weakly crystallized gadolinium oxide along with magnetic iron oxide (Figure 3, curves 4 and 5; Table 1, S8 and S9). Most parts of the samples are X-ray amorphous. So, the average crystallite size calculation of identified phases will not sufficiently correct for the characterization of the S8 and S9. In addition, it should be noted that this temperature is insufficient for the synthesis of mixed oxides GdFeO_3_ and Gd_3_FeO_6_.

**Figure 4:**
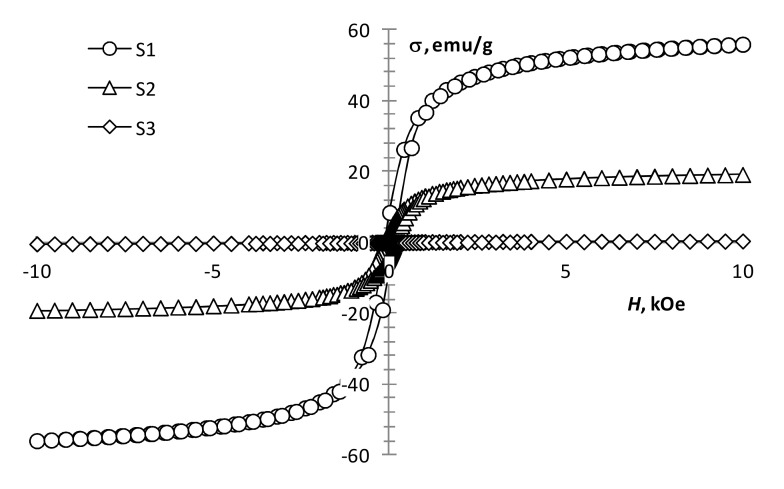
Magnetization loops of samples S1, S2, S3.

**Figure 5:**
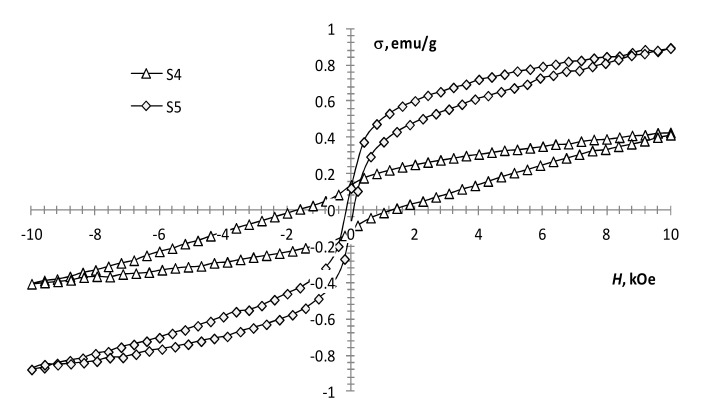
Magnetization loops of samples S4, S5.

The performed magnetic measurements showed that pyrolysis temperature affects the magnetic properties of the pyrolysis products of precursor 1 (Table 1, Figure 4). The magnetization of the samples reduces with an increase in pyrolysis temperature. However, it remains significant up to 450 °C (Figure 4, S1 and S2), which is characteristic of the presence of a ferrimagnetic phase [42]. At 550 °C, the magnetization decreases sharply, while the coercivity increases (Figure 4, S3; Figure 5, S4), which is typical for hematite [43]. This confirms the transition of the ferrimagnetic phase (magnetite or maghemite) to hematite, wherein changing the annealing time insignificantly affects the magnetic properties of the samples.

**Figure 6:**
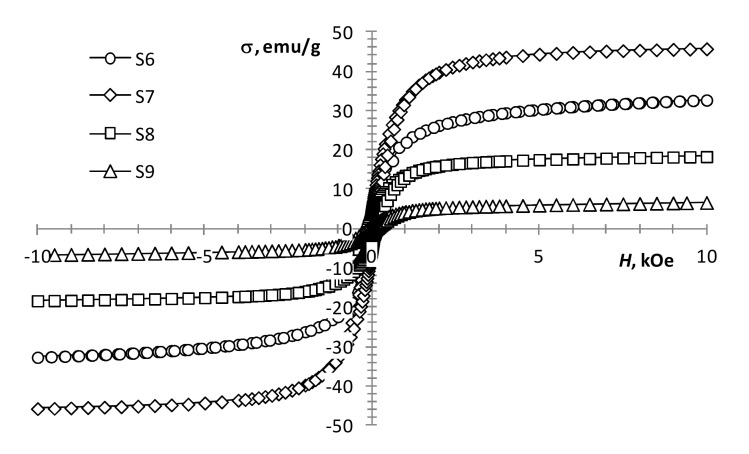
Magnetization loops of samples S6–S9.

With an increase in the gadolinium content, the change in the magnetization of the samples is not monotonic. At the same production conditions, the presence of a small amount of gadolinium (0.5 mol %) in precursors 1 and 2 mixture (Figure 5, S4 and S5) results in a decrease in coercivity and an increase in the magnetization of the sample. This may indicate the presence of some ferrimagnetic material in the sample, which is not detected by XRD analysis. A subsequent increase in the content of gadolinium to 2.5 mol % leads to the magnetization growth of the samples (Figure 5, S4 and S5; Figure 6, S6). With a further increase in the content of gadolinium, the magnetization of the products reduces (Figure 6, S7–S9). This can be explained by the presence of a large amount of non-magnetic gadolinium oxide.

According to the results presented in the work [21], an increase in the gadolinium content in Gd-doped magnetite produced by co-precipitation leads to a monotonic decrease in the magnetization. However, according to our results, an increase in the gadolinium content from 0.5 to 12.5 mol % leads to an increase in magnetization (Table 1, S5–S7; Figure 5 and Figure 6). The discrepancy between the results can be explained by the difference in the width of the size distribution of the resulting nanoparticles produced by different methods. Even in the case of the same average size, the samples can contain a different proportion of superparamagnetic particles, which radically changes the value of the coercivity and makes it difficult to determine the value of the spontaneous magnetization of the material.

**Figure 7:**
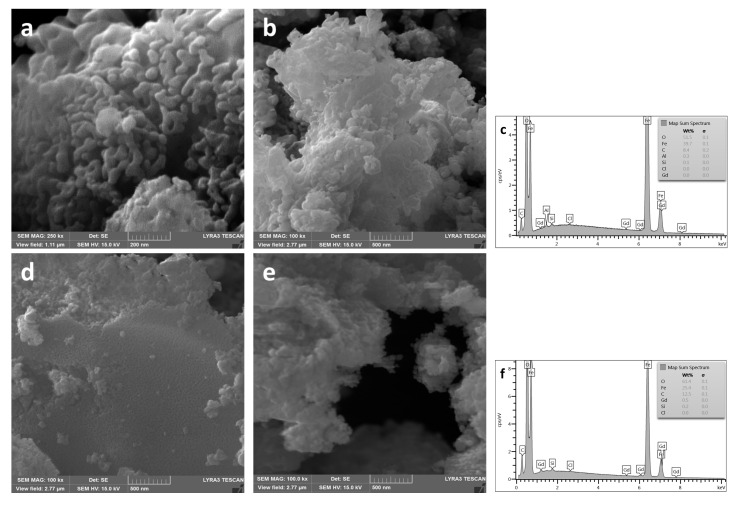
SEM images and EDX spectrum of powders produced at 550 °C: (**a**–**c**)—S4 (undoped Fe_2_O_3_); (**d**–**f**)—S5 (0.5 mol % Gd-doped Fe_2_O_3_).

The results of microscopic studies of S4 and S5 showed (Figure 7a,b,d,e) that hematite powders consist of elongated particles, the maximum length of which, in sample S4, reaches ~250 nm, but in sample S5 reaches ~100 nm at a width of ~30 nm. Therefore, the high values of H_c_ obtained for these samples may be associated with anisotropic structures that occur during hematite formation [44]. In the case of the sample doped with gadolinium (Figure 7d,e; Table 1, S5), the less pronounced anisotropy of α-Fe_2_O_3_ particles and the higher content of the magnetic phase do not lead to such a significant increase in H_c_. The elemental composition of the studied samples is given on the EDX spectra (Figure 7c,f). The spectrum presented in Figure 7f confirms the presence of gadolinium in 0.5 mol % Gd-doped Fe_2_O_3_ sample.

## 4. Conclusions

In the framework of research, the possibilities of the EPM for producing Gd-doped magnetic nanopowders have been demonstrated. According to the results of XRD analysis of samples produced by pyrolysis of an iron containing precursor at a temperature range of 350–550 °C, the complete magnetite-to-hematite phase transformation occurs at a temperature of 550 °C. However, according to magnetic measurements, residual amounts of the magnetic phase are still present in the sample produced at this temperature. It was shown that the presence of more than 2.5 mol % gadolinium valerate as gadolinium additive in the iron-containing precursor inhibits the magnetite-to-hematite transformation process at a pyrolysis temperature of the mixed precursor of 550 °C. Compared to widely used synthesis methods, magnetic properties of powders produced by EPM differ. This stimulates further detailed research, which can be useful for expanding the possibilities of application of the produced objects, especially in biomedicine.

## Figures and Tables

**Table 1:** XRD analysis and magnetic measurement results of produced samples.

Sample (S)	Gd Content, mol %	Preparation Conditions		X-Ray Phase Analysis Results		Magnetic Measurement Results	
		T, °C	t_anneal_, min	Phase Composition	*d*, nm	Magnetization at 10 kOe, emu/g	Coercivity H_c_, Oe
S1	0	350	30	Fe_3_O_4_/γ-Fe_2_O_3_ Admixture phase: α-Fe_2_O_3_	23 –	55.9	127
S2	0	450	30	Fe_3_O_4_/γ-Fe_2_O_3_ α-Fe_2_O_3_	36 39	19.2	144
S3	0	550	30	α-Fe_2_O_3_	45	0.40	1331
S4	0		60	α-Fe_2_O_3_	50	0.42	1481
S5	0.5			α-Fe_2_O_3_	45	0.89	120
S6	2.5			α-Fe_2_O_3_ Fe_3_O_4_/γ-Fe_2_O_3_	45 30	32.7	87
S7	12.5			Fe_3_O_4_/γ-Fe_2_O_3_	21	45.7	84
S8	50			Fe_3_O_4_/γ-Fe_2_O_3_, Gd_2_O_3_, Fe_2_GdO_4_	– –	18.3	134
S9	75			Fe_3_O_4_/γ-Fe_2_O_3_, Gd_2_O_3_, Fe_2_GdO_4_	– –	6.7	120
